# Supplementary material for: A cell line model for the study of CD4-negative HIV-1 infection and latent virus reservoirs
Source: PLoS One. 2026 Nov 12;21(11):e0361655. doi: 10.1371/journal.pone.0361655 (PMC13759070; doi:10.1371/journal.pone.0361655)
Supplement: Supplementary file 1 [file pone.0361655.s001.pdf]

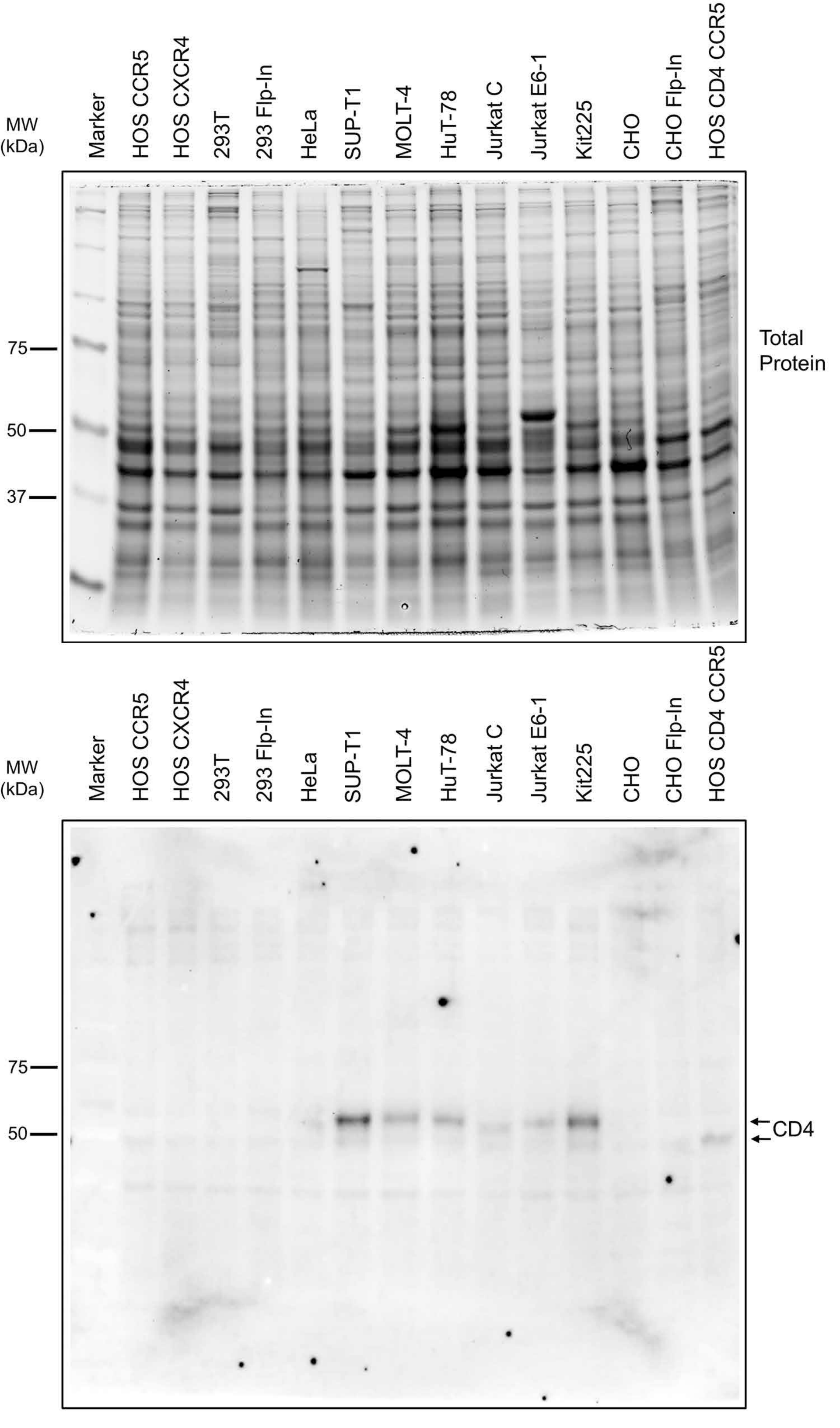
MW (kDa)
Marker
HOS CCR5
HOS CXCR4
293T
293 Flp-In
HeLa
SUP-T1
MOLT-4
HuT-78
Jurkat C
Jurkat E6-1
Kit225
CHO
CHO Flp-In
HOS CD4 CCR5
75
50
37
Total Protein
MW (kDa)
Marker
HOS CCR5
HOS CXCR4
293T
293 Flp-In
HeLa
SUP-T1
MOLT-4
HuT-78
Jurkat C
Jurkat E6-1
Kit225
CHO
CHO Flp-In
HOS CD4 CCR5
75
50
CD4
