# Supplementary material for: A cell line model for the study of CD4-negative HIV-1 infection and latent virus reservoirs
Source: PLoS One. 2026 Nov 12;21(11):e0361655. doi: 10.1371/journal.pone.0361655 (PMC13759070; doi:10.1371/journal.pone.0361655)
Supplement: Supplementary file 2 [file pone.0361655.s002.pdf]

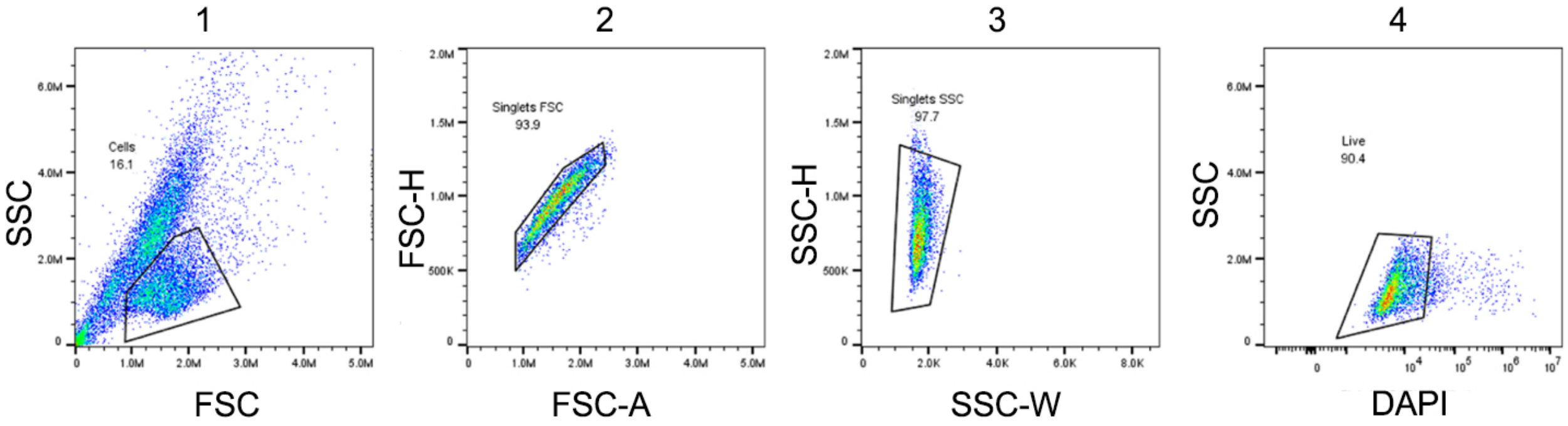

S1_Fig. Gating strategy for flow cytometry.
(1) FSC vs SSC (2) Single cell gate 1. (3) Single cell gate 2. (4) viability gate (DAPI negative cells).
