# Supplementary material for: A cell line model for the study of CD4-negative HIV-1 infection and latent virus reservoirs
Source: PLoS One. 2026 Nov 12;21(11):e0361655. doi: 10.1371/journal.pone.0361655 (PMC13759070; doi:10.1371/journal.pone.0361655)
Supplement: Supplementary file 3 [file pone.0361655.s003.pdf]

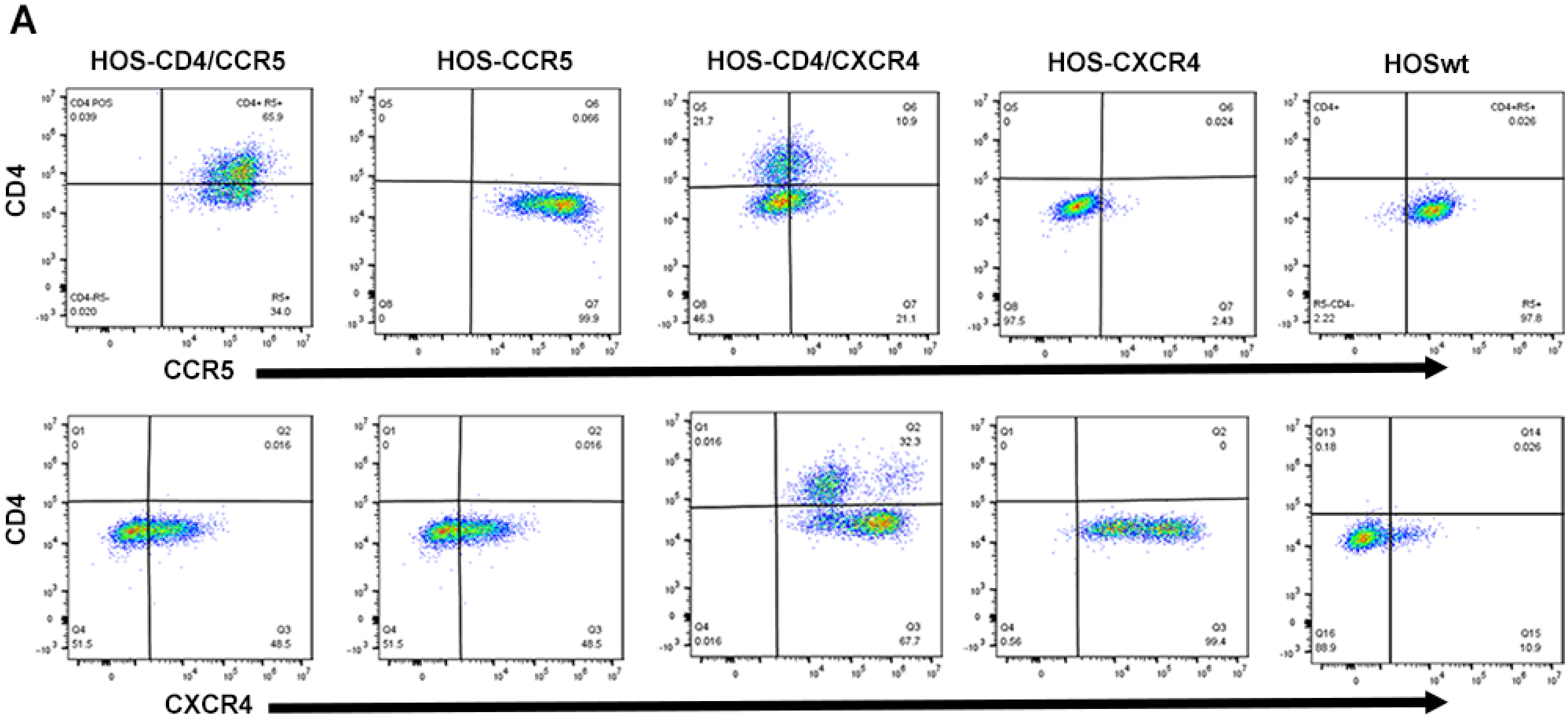

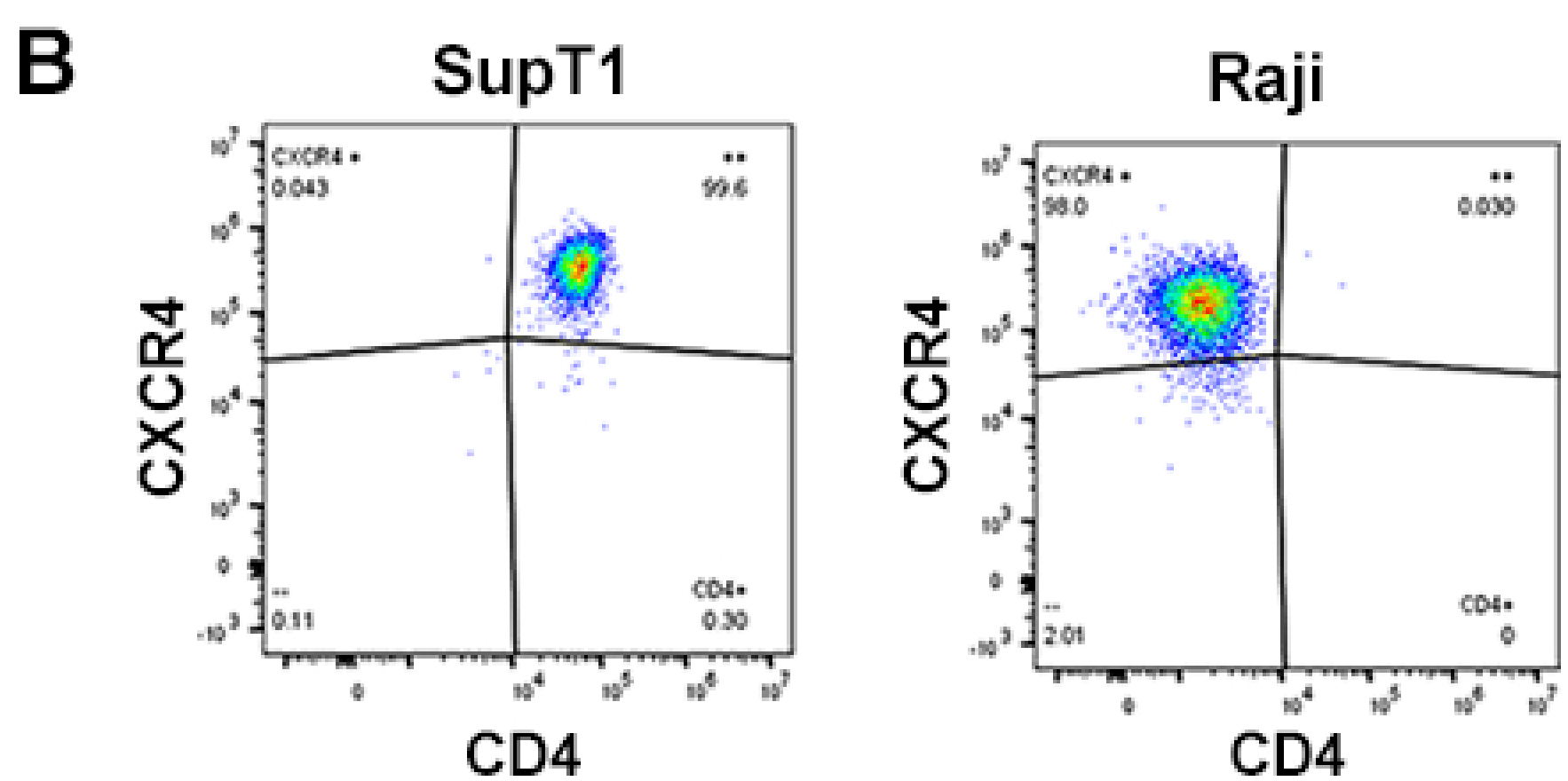

S2 Fig. Extended data for Table 1 and Figure 1D

(A) Expression of CD4, CXCR4 and CCR5 was characterized in all HOS derivatives as described in the Methods section. The percent positive for each marker is listed in Table 1.

(B) CD4 expression was determined in SupT1 (positive control) and the Raji B cell line (negative control) as supporting evidence for the qPCR data in Figure 1D. Both cell lines are strongly postitive for CXCR4 expression.
