# Supplementary material for: A cell line model for the study of CD4-negative HIV-1 infection and latent virus reservoirs
Source: PLoS One. 2026 Nov 12;21(11):e0361655. doi: 10.1371/journal.pone.0361655 (PMC13759070; doi:10.1371/journal.pone.0361655)
Supplement: Supplementary file 4 [file pone.0361655.s004.pdf]

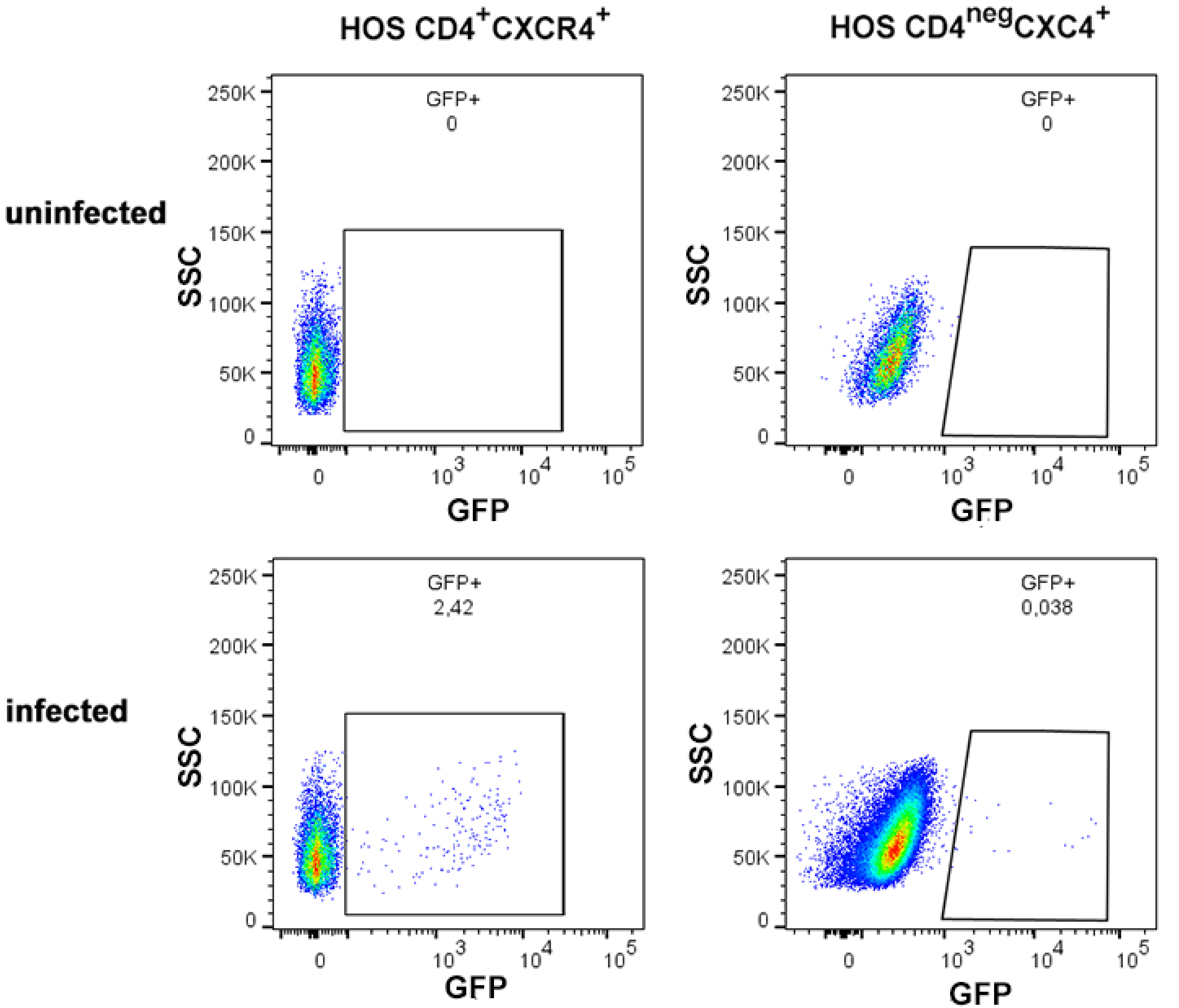

S3 Fig. Extended data for Figure 2

CD4 positive or negative CXCR4-HOS cells were infected with GFP-expressing, HXB2-enveloped HIV as described for Figure 2. Instead of immunofluorescence, cells were harvested using accutase and analyzed by flow cytometry. Infected cells were gated using unifected cells.
